# Cause of Angular Distortion in Fusion Welding: Asymmetric Cross-Sectional Profile along Thickness

**DOI:** 10.3390/ma12010058

**Published:** 2018-12-24

**Authors:** Deqiao Xie, Jianfeng Zhao, Huixin Liang, Shuang Liu, Zongjun Tian, Lida Shen, Changjiang Wang

**Affiliations:** 1 College of Mechanical and Electrical Engineering, Nanjing University of Aeronautics and Astronautics, Nanjing 210016, China; dqxie@nuaa.edu.cn (D.X.); hxliang@nuaa.edu.cn (H.L.); shuangliu@nuaa.edu.cn (S.L.); tianzj@nuaa.edu.cn (Z.T.); ldshen@nuaa.edu.cn (L.S.); 2 Nanjing Institution of Advanced Laser Technology, Nanjing 210038, China; 3 Department of Engineering and Design, University of Sussex, Sussex House, Brighton BN1 9RH, UK; C.J.Wang@sussex.ac.uk

**Keywords:** angular distortion, cross-section, weld seam, bending moment, welding

## Abstract

Angular distortion is a common problem in fusion welding, especially when it comes to thick plates. Despite the fact that various processes and influencing factors have been discussed, the cause of the angular distortion has not been clearly revealed. In this research, the asymmetry of cross-sectional profile along thickness is considered of great importance to the angular distortion. A theoretical model concerning the melting-solidification process in fusion welding was established. An expression of the angular distortion was formulated and then validated by experiments of laser welding 316L stainless steel. The results show that the asymmetric cross-sectional profile is a major contributory factor towards the angular distortion mechanism. The asymmetry of cross-section profile along thickness causes the difference between two bending moments in the lower and upper parts of the joint. This is the difference that drives the angular distortion of the welded part. Besides, the asymmetry of cross-section profile is likely to be influenced by various processes and parameters, thereby changing the angular distortion.

## 1. Introduction

Fusion welding is a commonly used joint technology for manufacturing various metal structures, particularly within the aeronautics, automotive, ships, and architecture [1,2,3,4,5]. However, fusion welding is usually accompanied by unavoidable shrinkage and undesired distortion, which can decrease the precision of the welded part [6,7].

According to Zhou, angular distortion is a common distortion in fusion welding [8]. He concluded several factors were related including plate thickness, total heat input, amount of weld metal used, and groove shape. Wang et al. [9] investigated that angular distortion could be distinctly changed by heat input and plate thickness. Okano et al. [10] and Tian et al. [11] observed that angular distortion could be decreased when the heat input was larger. However, Adamczuk et al. [12] discovered that angular distortion may not be affected by heat input. In terms of the amount of weld metal, angular distortion was more dominant in multi-pass welding than that in single-pass welding [13]. Cai et al. [14] revealed that angular distortion in butt-joint welding of V-groove items was larger than that of X-groove.

Researchers also explored other processes and parameters that could change angular distortion in welding. Zhang et al. [15] found a nonlinear relationship between angular distortion and arc distance. Tseng and Chou [16] found that angular distortion increased with the proportion of nitrogen in the shielding gas for welding austenitic stainless steel. The austenitic matrix is beneficial for reducing angular distortion. Thermal expansion coefficient is also recognized as a significant parameter for accurate predictions of angular distortion [17]. Park et al. [18] found that pre-tension stress loaded in the transversal direction diminished welding angular distortion by 60%. Kelly et al. [19] stated that the laser-arc hybrid welding obtained less distortion compared with arc welding. Rong et al. [20] applied a magnetic field in laser welding. The welding distortion was decreased by 26.56%. Mochizuki et al. [21] found that reverse-side TIG heating ahead of MIG welding was able to reduce angular distortion. Michaleris [22] pointed out several ways to reduce angular distortion including presetting, restraint, and line heating.

Researchers have shown that the factors are various and complicated which could change the angular distortion in welding. But the following remain unknown: (1) How to judge the directions of angular distortion. (2) How do the factors directly change and to what extent can such factors change the angular distortion? (3) Whether the angular distortion can be expressed in a calculation. This study aims to reveal the mechanism of angular distortion in fusion welding and how the welding processes and factors influence the angular distortion In this study, a theoretical model concerning the melting-solidification process in fusion welding will be established. An expression of the angular distortion will be formulated and then validated by laser welding 316L austenitic stainless steel. Based on the research, the angular distortion will be controlled and adjusted with a quantitative guideline.

## 2. Theoretical Model

**Figure 1:**
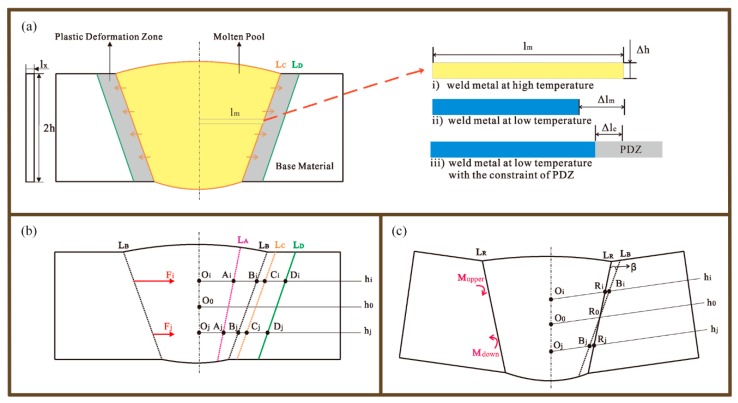
Theoretical model of angular distortion in fusion welding. (**a**) Schematic diagram of the weld seam cross-section when melting and cooling. (**b**) The constraining forces at different heights. (**c**) The bending moments *M_upper_* and *M_down_* and the angular distortion *β*.

A theoretical model of the angular distortion in fusion welding is demonstrated in Figure 1. Figure 1a shows the schematic diagram of a weld seam cross-section when the metal was melted. Adequate high energy input, like arc, laser, and electron beam, heated the metal to form a molten pool. The metal in the molten pool was expanded, resulting in the compression of the base material adjacent to boundary of the molten pool. The base material heat-affected by the molten pool was easily plastically deformed [23,24]. The plastic deformation zone (PDZ) can be assumed between the lines L_C_ and L_D_.

After the heat input was removed, the molten pool cooled down and then shrunk. However, the shrinkage had to be restricted by the PDZ. The right region of Figure 1a illustrates this phenomenon in a specific way. The length of a rod extracted from the molten pool was *l_m_* when the metal was at a high temperature. If there was no constraint, the rod could shrink to *Δl_m_* after it cooled. However, the material at the PDZ had been compressed before. The PDZ had to be stretched in a reverse direction for the shrinkage of weld metal. There would be an equilibrium between the rod and the PDZ. Finally, the rod had a shrinkage of *Δl_c_* with the constraint of PDZ. An interactive constraining force can be assumed between the rod and PDZ [25].

Since the weld metal in the cross-section can be divided into a mass of rods at different heights, there would be associated constraining forces at associated heights between weld metal and the PDZ. Figure 1b shows two typical constraining forces at heights of *h_i_* and *h_j_*. The *h_i_* and *h_j_* represent the heights above and down the center line *h_0_* respectively. Line L_A_ depicted the ideal boundary of the weld metal if there was no constraint. Line *L_B_* was the actual boundary of the weld metal with the constraint of the PDZ if angular distortion was not generated. The rods at different heights were assumed to be ideal elastic bodies. According to Hooke’s law, the constraining force *F_i_* is expressed as below.

(1) $$F_{i}=\frac{\bar{A_{i}C_{i}}-\bar{B_{i}C_{i}}}{\bar{O_{i}C_{i}}}\cdot E\cdot \Delta h\cdot l_{x}=\frac{\bar{A_{i}B_{i}}}{\bar{O_{i}C_{i}}}\cdot E\cdot \Delta h\cdot l_{x}$$

(2) $$\bar{A_{i}C_{i}}=(\alpha \cdot \Delta T+\beta_{pt})\cdot \bar{O_{i}C_{i}}$$

*E* is the Young’s modulus of weld metal. $\Delta h\cdot l_{x}$ represents the cross-section area of the rod. The ideal shrinkage $\bar{A_{i}C_{i}}$ is composed of thermal shrinkage α⋅ΔT and phase transformation shrinkage *β_pt_* [26]. The *α* is thermal expansion coefficient of the weld metal, while *∆T* represents the temperature difference between solidus temperature and room temperature.

The $\bar{B_{i}C_{i}}$ represents the length after equilibrium of weld metal’s shrinkage and the PDZ’s stretch. The PDZ was more difficult to be stretched, because it was colder and had been severely compressed before. Wang [24] reported a tiny backward stretch of the PDZ after an obvious compressive plastic strain. Therefore, we assume that the PDZ dominates the equilibrium, and the $\bar{B_{i}C_{i}}$ reflects the capacity of reverse stretch of the PDZ. The relationship between $\bar{B_{i}C_{i}}$ and PDZ is assumed to be:

(3) $$\bar{B_{i}C_{i}}=k\cdot \bar{C_{i}D_{i}}$$

*k* is the coefficient about reverse stretch of PDZ. It may be determined by the welded metal properties like thermal expansion and plasticity. So, the constraining forces *F_i_* and *F_j_* can be expressed more specifically as below:

(4) $$\begin{array}{l} F_{i}=\frac{(\alpha \cdot \Delta T+\beta_{pt})\cdot \bar{O_{i}C_{i}}-k\cdot \bar{C_{i}D_{i}}}{\bar{O_{i}C_{i}}}\cdot E\cdot \Delta h\cdot l_{x}\\ =(\alpha \cdot \Delta T+\beta_{pt}-k\cdot \frac{\bar{C_{i}D_{i}}}{\bar{O_{i}C_{i}}})\cdot E\cdot \Delta h\cdot l_{x} \end{array}$$

(5) $$\begin{array}{l} F_{j}=\frac{(\alpha \cdot \Delta T+\beta_{pt})\cdot \bar{O_{j}C_{j}}-k\cdot \bar{C_{j}D_{j}}}{\bar{O_{j}C_{j}}}\cdot E\cdot \Delta h\cdot l_{x}\\ =(\alpha \cdot \Delta T+\beta_{pt}-k\cdot \frac{\bar{C_{j}D_{j}}}{\bar{O_{j}C_{j}}})\cdot E\cdot \Delta h\cdot l_{x} \end{array}$$

(6) $$M_{upper}=\sum_{i=0}^{h}F_{i}\cdot h_{i}$$

(7) $$M_{down}=\sum_{j=0}^{h}F_{j}\cdot h_{j}$$

As expressed in Formulas (6) and (7), the bending moments *M_upper_* and *M_down_* derived from the constraining forces in the upper and lower parts of the cross-section, respectively. If the *M_upper_* was different from *M_down_*, the base material was driven to rotate, forming an angular distortion *β*. The final boundary of cross-section *L_R_* was depicted in Figure 1d. The *F_i_* and *F_j_* should be amended as follows:

(8) $$F_{i}=(\alpha \cdot \Delta T+\beta_{pt}-\frac{k\cdot \bar{C_{i}D_{i}}-\sqrt{{\left(\bar{O_{i}R_{i}}-\bar{O_{0}R_{0}}\right)}^{2}+h_{i}^{2}}\cdot \beta }{\bar{O_{i}C_{i}}})\cdot E\cdot \Delta h\cdot l_{x}$$

(9) $$F_{j}=(\alpha \cdot \Delta T+\beta_{pt}-\frac{k\cdot \bar{C_{j}D_{j}}+\sqrt{{\left(\bar{O_{j}R_{j}}-\bar{O_{0}R_{0}}\right)}^{2}+h_{j}^{2}}\cdot \beta }{\bar{O_{j}C_{j}}})\cdot E\cdot \Delta h\cdot l_{x}$$

(10) $$\bar{O_{i}C_{i}}\approx \bar{O_{i}R_{i}}$$

(11) $$\bar{O_{j}C_{j}}\approx \bar{O_{j}R_{j}}$$

The bending moment *M_upper_* would be equal to *M_down_* finally.

(12) $$\sum_{i=0}^{h}F_{i}\cdot h_{i}=\sum_{j=0}^{h}F_{j}\cdot h_{j}$$

(13) $$\begin{array}{l} \sum_{i=0}^{h}(\alpha \cdot \Delta T+\beta_{pt}-\frac{k\cdot \bar{C_{i}D_{i}}-\sqrt{{\left(\bar{O_{i}R_{i}}-\bar{O_{0}R_{0}}\right)}^{2}+h_{i}^{2}}\cdot \beta }{\bar{O_{i}R_{i}}})\cdot E\cdot \Delta h\cdot l_{x}\cdot h_{i}\\ =\sum_{j=0}^{h}(\alpha \cdot \Delta T+\beta_{pt}-\frac{k\cdot \bar{C_{j}D_{j}}-\sqrt{{\left(\bar{O_{j}R_{j}}-\bar{O_{0}R_{0}}\right)}^{2}+h_{j}^{2}}\cdot \beta }{\bar{O_{j}R_{j}}})\cdot E\cdot \Delta h\cdot l_{x}\cdot h_{j} \end{array}$$

So, the angular distortion *β* can be formulated as below.

(14) $$\beta =k\cdot \frac{\sum_{j=0}^{h}\frac{\bar{C_{j}D_{j}}\cdot h_{j}}{\bar{O_{j}R_{j}}}-\sum_{i=0}^{h}\frac{\bar{C_{i}D_{i}}\cdot h_{i}}{\bar{O_{i}R_{i}}}}{\sum_{j=0}^{h}\frac{\sqrt{{\left(\bar{O_{j}R_{j}}-\bar{O_{0}R_{0}}\right)}^{2}+h_{j}^{2}}}{\bar{O_{j}R_{j}}}\cdot h_{j}+\sum_{i=0}^{h}\frac{\sqrt{{\left(\bar{O_{i}R_{i}}-\bar{O_{0}R_{0}}\right)}^{2}+h_{i}^{2}}}{\bar{O_{i}R_{i}}}\cdot h_{i}}$$

(15) $$\beta =k\cdot \bar{CD}\cdot Exp(A)$$

(16) $$Exp(A)=\frac{\sum_{j=0}^{h}\frac{h_{j}}{\bar{O_{j}R_{j}}}-\sum_{i=0}^{h}\frac{h_{i}}{\bar{O_{i}R_{i}}}}{\sum_{j=0}^{h}\frac{\sqrt{{\left(\bar{O_{j}R_{j}}-\bar{O_{0}R_{0}}\right)}^{2}+h_{j}^{2}}}{\bar{O_{j}R_{j}}}\cdot h_{j}+\sum_{i=0}^{h}\frac{\sqrt{{\left(\bar{O_{i}R_{i}}-\bar{O_{0}R_{0}}\right)}^{2}+h_{i}^{2}}}{\bar{O_{i}R_{i}}}\cdot h_{i}}$$

Zhou’s study [27] showed isothermal curve parallel to the cross-section profile in laser welding. So, $\bar{C_{i}D_{i}}$ and $\bar{C_{j}D_{j}}$ in Formula (14) are regarded to be the same as $\bar{CD}$. The other part in Formula (14) was replaced by Exp(A), as shown in Formulas (15) and (16). It can be seen that the angular distortion *β* is decomposed by three parts: weld metal properties, length of plastic deformation zone, and cross-section profile of weld seam. Here, $\bar{CD}$ reflects the length of plastic deformation zone (PDZ) that caused by expansion of the molten pool in welding. The *k* is determined by the weld material properties. Exp(A) reflects the difference between upper and down parts of weld seam cross-section, i.e., the asymmetry of the cross-sectional profile along thickness. Zhou was very close to this finding because he mentioned that angular distortion was caused by temperature differences between the top and bottom surfaces of the plate [8].

## 3. Experimental

Laser has been widely utilized in automatic welding for automotive, electronics, and aircrafts [28]. Laser welding is an attractive option because laser is stable and repeatable. In this experiment, austenitic stainless steel AISI 316L (EN 1.4404) was used, as it is a commonly used austenitic stainless steel within marine, energy, aerospace, and medical environments due to its excellent strength and corrosion resistance performances [29,30].

**Figure 2:**
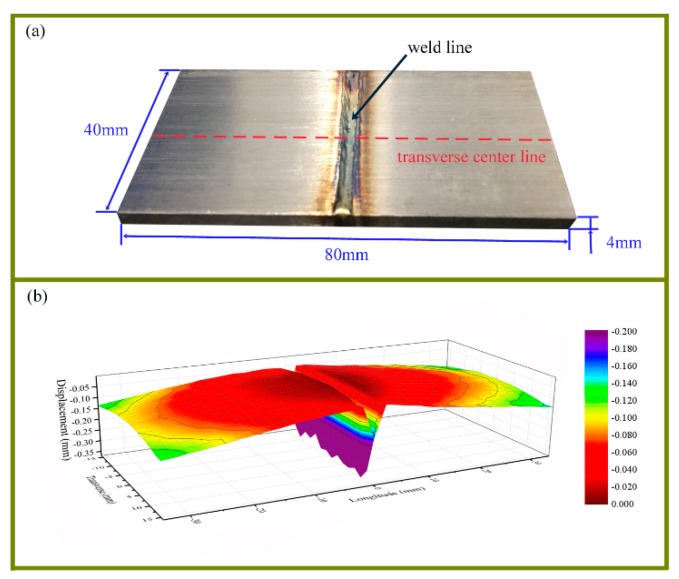
The welded 316L stainless steel and morphology of the bottom surface of case S1. (**a**) The welded part. (**b**) The morphology of the bottom surface.

The experiment was performed on a laser welding system consisting of a 6 kW TRUMPF disk laser (Trumpf Co. ltd, Ditzingen, Germany) with working wavelength of 1064 nm, a KUKA robot (Kuka Robot, Augsburg, Germany), and a PRECITEC welding head (Precitec GmbH, Gaggenau, Germany). The molten pool was shielded by argon gas. The 80 mm × 40 mm × 4 mm AISI 316L plates (Haocheng, Shanghai, China) were welded, as shown in Figure 2a. The parts were welded at a velocity of 15 mm/s.

The distortion of the substrate was measured by a coordinate measuring machine from Leader Metrology Inc., MD, America. The displacement data of a 2 mm × 2 mm dot matrix on the back of the substrate was obtained. The measured data were used to create 3D contour plot with Origin Software, so as to demonstrate the distortion intuitively. Figure 2b shows software morphology reconstruction of the bottom surface of case S1. It depicts a symmetrical and downward distortion of the butt-joint laser welding. The data at transverse center line were also extracted.

In order to investigate the cross-sectional profile, the cases were cut via wire-Electrical Discharging Machining(EDM) (Huafang, Hangzhou, China)EDM. The microstructure was observed on an OLYMPUSGX71 optical microscope (Olympus Corporation, Tokyo, Japan) after using the etchant of 5g CuCl_2_ +100 mL HCl + 100 mL CH_3_CH_2_OH.

## 4. Results and Discussion

### 4.1. The Cross-Sectional Profiles of S1–S6 Cases

**Figure 3:**
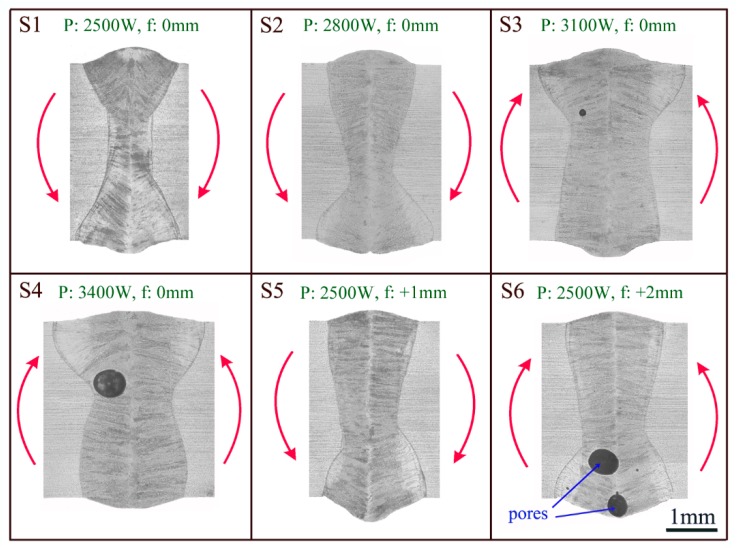
The cross-sectional profile of cases S1–S6. P represents laser power, while f means focus offset. The red arrows show actual angular distortion directions.

Based on Formulas (15) and (16), we can quickly judge that if the down section of profile was broader than the upper section, angular distortion *β* in Formula (14) would be negative, and vice versa. The broader profile in down section meant that $\bar{O_{j}R_{j}}$ was generally longer than $\bar{O_{i}R_{i}}$, resulting in a negative angular distortion like case S1 in Figure 3.

**Figure 4:**
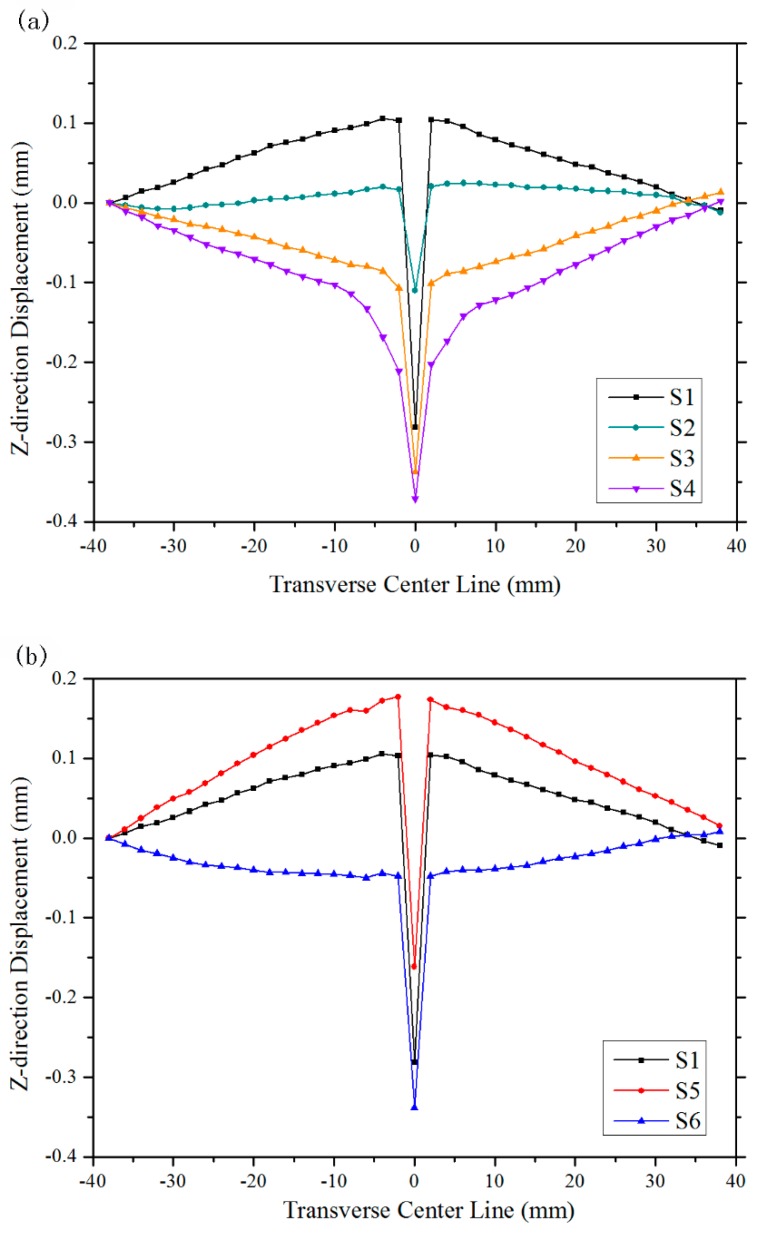
The Z-direction displacements of transverse center line of cases S1-S6. (**a**) Z-direction displacements of various laser powers. (**b**) Z-direction displacements of various focus offsets.

Figure 3 also reveals that the upper section became wider as laser power increased, as shown by cases S1 S2, S3, and S4. Hence, it can be predicted that the angular distortion would become gradually upward. The estimation was later validated by Figure 4a, which shows Z-direction displacements of transverse center lines of cases S1–S4. It can be inferred that the asymmetric cross-sectional profile along thickness is a major contributory factor towards angular distortion mechanism.

When the focus offset increased, as seen in S1, S5, and S6, the down section profile became larger. So, case S5 obtained a more obvious downward angular distortion than S1. But for S6, the excessive focus offset tended to decrease the density of the laser power, which led to insufficient flow in the molten pool. As a result, the pores were hard to flee. The big pores at the lower section released the constraint between weld metal and the PDZ to a certain extent. The bending moment of upper section *M_upper_* could drive base material to bend upward, as depicted in Figure 4b.

### 4.2. Calculation of the Angular Distortion

**Figure 5:**
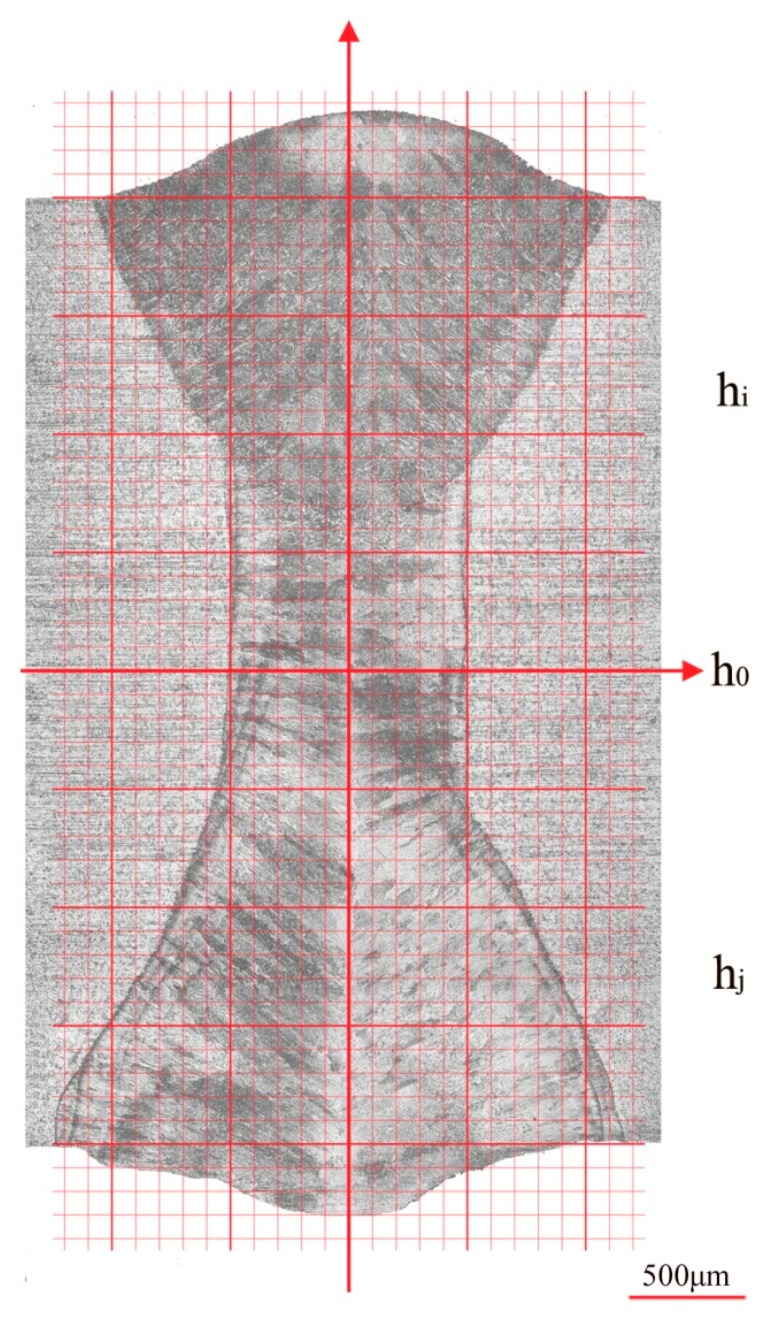
The profile measurement of case S1 by using a cartesian coordinate system.

In order to obtain the data of profile asymmetry, a cartesian coordinate system was established upon the cross-section image, as shown in Figure 5. The lengths of the $\bar{O_{i}R_{i}}$ at different heights *h_i_* above the center line *h_0_* were measured, as well as lengths of the $\bar{O_{j}R_{j}}$ at different heights *h_j_* down *h_0_*. Table 1 shows the cross-section profile of case S1.

The profiles of cases S2–S6 were shown in supplementary Table A1, Table A2, Table A3, Table A4 and Table A5 (refer to Appendix A). Table 2 exhibits the calculated and $\bar{CD}$ and Exp(A), as well as the measured angular distortions of cases S1–S6. The nonlinear Z-direction displacement near weld line revealed the PDZ, for example the region from −12 mm to 12 mm on the transverse center line of case S6. The regions from −30 mm to −20 mm and from 20 mm to 30 mm of transverse center line are thought to reflect the real angular distortion of the cases, because the connection lines lie on nearly same straight lines. *β_average_* was the average value of *β_left_* and *β_right_*.

**Figure 6:**
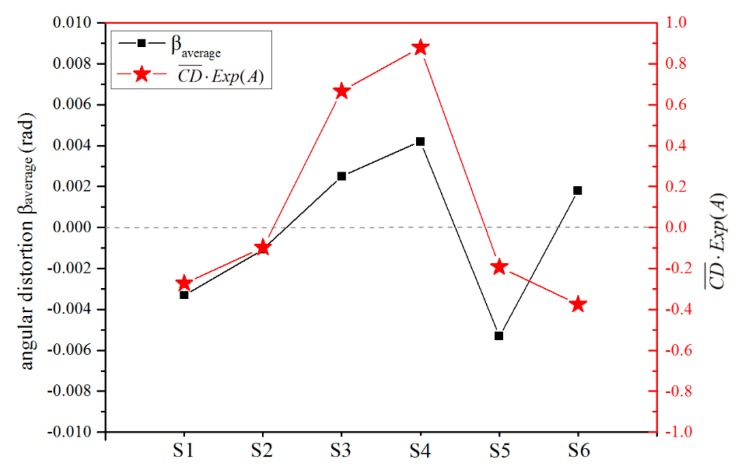
The measured angular distortions and calculated $\bar{CD}\cdot Exp(A)$ of cases S1–S6.

Figure 6 demonstrates the relationship between calculated $\bar{CD}\cdot Exp(A)$ and measured angular distortions of cases S1–S6. The calculated $\bar{CD}\cdot Exp(A)$ verified angular distortion directions of cases S1–S5 quite well. However, the pores in case S6 distinctly changed the predicted direction. The errors between the calculated and the measured values may come from the undesired pores, the inaccurate estimation of the PDZ length $\bar{CD}$ and the inhomogeneous microstructure of the weld seam, which can affect the elastic and plastic performances. Further studies are required for more accurate calculation of angular distortion.

## 5. Conclusions

Previous studies have shown that angular distortion can be influenced by various processes and parameters. However, the cause of angular distortion remains unclear. In this study, the influence of cross-sectional profile on the angular distortion is firstly valued. The authors established a theoretical model to illustrate the melting-solidification process in laser welding, and then formulated an expression of the angular distortion. The calculation process of angular distortion was shown based on cases S1–S6. The experimental results matched the theoretical findings well. The conclusions are as follows:

(1) The asymmetry of the cross-sectional profile causes the difference between bending moments *M_upper_* and *M_down_*, thereby causing the angular distortion. The angular distortion direction depends on whether the broader profile is at upper or at down section of the weld beam.

(2) The angular distortion seems to be related to weld metal properties, length of plastic deformation zone and, most important, the asymmetry of the cross-sectional profile along thickness.

(3) The various processes and parameters can change the asymmetry of the cross-sectional profile, which will change the angular distortion. It is promising to minimize the angular distortion by decreasing the difference between the upper and lower profile of the weld seam.

(4) The pores in the weld seam can distinctly change the direction and value of predicted welding angular distortion.

## Figures and Tables

**Table 1:** The coordinate values of the cross-sectional profile of case S1. (Unit: mm).

Height Upper (h_i_)	Left Length	Right Length	Average Length (O_i_R_i_)	Height Down (h_j_)	Left Length	Right Length	Average Length (O_j_R_j_)
0.1	0.50	0.50	0.50	0.1	0.51	0.50	0.51
0.2	0.50	0.50	0.50	0.2	0.53	0.50	0.52
0.3	0.50	0.50	0.50	0.3	0.54	0.50	0.52
0.4	0.50	0.50	0.50	0.4	0.55	0.50	0.53
0.5	0.50	0.50	0.50	0.5	0.56	0.50	0.53
0.6	0.52	0.52	0.52	0.6	0.60	0.50	0.55
0.7	0.54	0.52	0.53	0.7	0.63	0.60	0.62
0.8	0.54	0.52	0.53	0.8	0.67	0.64	0.66
0.9	0.53	0.53	0.53	0.9	0.70	0.70	0.70
1.0	0.56	0.52	0.54	1.0	0.75	0.75	0.75
1.1	0.66	0.68	0.67	1.1	0.78	0.80	0.79
1.2	0.72	0.74	0.73	1.2	0.83	0.83	0.83
1.3	0.77	0.80	0.79	1.3	0.90	0.90	0.90
1.4	0.82	0.84	0.83	1.4	0.97	0.95	0.96
1.5	0.87	0.89	0.88	1.5	1.02	0.98	1.00
1.6	0.92	0.92	0.92	1.6	1.08	1.04	1.06
1.7	0.98	0.96	0.97	1.7	1.15	1.09	1.12
1.8	1.02	1.02	1.02	1.8	1.22	1.12	1.17
1.9	1.06	1.08	1.07	1.9	1.24	1.14	1.19
2.0	1.10	1.10	1.10	2.0	1.24	1.16	1.20
0	0.50	0.50	0.50				

**Table 2:** Calculated $\bar{CD}$ and Exp(A), as well as the measured angular distortions of cases S1–S6.

Case	$\bar{CD}$	Exp(A)	$\bar{CD}\cdot Exp(A)$	β_left_	β_right_	β_average_
S1	4.80	−5.660× 10^−2^	−2.72× 10^−1^	–3.740× 10^−3^	−2.870× 10^−3^	–3.310× 10^−3^
S2	6.61	−1.474× 10^−2^	−9.74× 10^−2^	–1.340× 10^−3^	−7.700× 10^−4^	–1.060× 10^−3^
S3	8.77	7.598× 10^−2^	6.66× 10^−1^	2.180× 10^−3^	2.810× 10^−3^	2.500× 10^−3^
S4	10.80	8.141× 10^−2^	8.79× 10^−1^	3.650× 10^−3^	4.750× 10^−3^	4.200× 10^−3^
S5	8.81	−2.189× 10^−2^	−1.93× 10^−1^	–5.450× 10^−3^	–5.140× 10^−3^	–5.300× 10^−3^
S6	8.61	−4.358× 10^−2^	−3.75× 10^−1^	1.540× 10^−3^	2.060× 10^−3^	1.800 × 10^−3^

## Appendix A

**Table A1:** Cross-sectional profile of case S2 weld seam. (Unit: mm).

Height Upper (h_i_)	Left Length	Right Length	Average Length (O_i_R_i_)	Height Down (h_j_)	Left Length	Right Length	Average Length (O_j_R_j_)
0.1	0.65	0.53	0.59	0.1	0.60	0.50	0.55
0.2	0.68	0.58	0.63	0.2	0.58	0.50	0.54
0.3	0.73	0.62	0.68	0.3	0.56	0.50	0.53
0.4	0.76	0.64	0.70	0.4	0.53	0.50	0.515
0.5	0.78	0.65	0.72	0.5	0.52	0.50	0.51
0.6	0.82	0.68	0.75	0.6	0.52	0.55	0.535
0.7	0.85	0.72	0.79	0.7	0.53	0.62	0.575
0.8	0.88	0.76	0.82	0.8	0.58	0.70	0.64
0.9	0.93	0.80	0.87	0.9	0.64	0.80	0.72
1.0	0.97	0.85	0.91	1.0	0.72	0.90	0.81
1.1	1.00	0.88	0.94	1.1	0.81	1.00	0.905
1.2	1.03	0.90	0.97	1.2	0.93	1.10	1.015
1.3	1.04	0.92	0.98	1.3	1.04	1.18	1.11
1.4	1.04	0.94	0.99	1.4	1.15	1.22	1.185
1.5	1.04	0.95	1.00	1.5	1.22	1.30	1.26
1.6	1.05	0.97	1.01	1.6	1.25	1.35	1.3.0
1.7	1.07	0.99	1.03	1.7	1.28	1.4	1.34
1.8	1.09	1.00	1.05	1.8	1.33	1.45	1.39
1.9	1.10	1.00	1.05	1.9	1.35	1.44	1.395
2.0	1.10	1.00	1.05	2.0	1.35	1.42	1.385
0	0.62	0.50	0.56				

**Table A2:** Cross-sectional profile of case S3 weld seam. (Unit: mm).

Height Upper (h_i_)	Left Length	Right Length	Average Length (O_i_R_i_)	Height Down (h_j_)	Left Length	Right Length	Average Length (O_j_R_j_)
0.1	0.95	0.96	0.955	0.1	0.98	0.98	0.98
0.2	0.90	0.96	0.93	0.2	1.00	1.00	1.00
0.3	0.88	0.98	0.93	0.3	1.02	1.00	1.01
0.4	0.88	1.00	0.94	0.4	1.04	1.00	1.02
0.5	0.87	1.00	0.935	0.5	1.05	1.02	1.04
0.6	0.90	1.04	0.97	0.6	1.08	1.04	1.06
0.7	0.98	1.08	1.03	0.7	1.10	1.08	1.09
0.8	1.05	1.16	1.105	0.8	1.12	1.10	1.11
0.9	1.13	1.25	1.19	0.9	1.13	1.12	1.13
1.0	1.25	1.33	1.29	1.0	1.13	1.13	1.13
1.1	1.32	1.46	1.39	1.1	1.15	1.15	1.15
1.2	1.41	1.55	1.48	1.2	1.16	1.17	1.17
1.3	1.47	1.60	1.535	1.3	1.17	1.18	1.18
1.4	1.52	1.68	1.60	1.4	1.18	1.20	1.19
1.5	1.58	1.74	1.66	1.5	1.2	1.21	1.21
1.6	1.62	1.77	1.695	1.6	1.21	1.22	1.22
1.7	1.66	1.82	1.74	1.7	1.23	1.23	1.23
1.8	1.69	1.82	1.755	1.8	1.26	1.24	1.25
1.9	1.70	1.82	1.76	1.9	1.26	1.24	1.25
2.0	1.70	1.8	1.75	2.0	1.26	1.20	1.23
0	0.96	1.00	0.98				

**Table A3:** Cross-sectional profile of case S4 weld seam. (Unit: mm).

Height Upper (h_i_)	Left Length	Right Length	Average Length (O_i_R_i_)	Height Down (h_j_)	Left Length	Right Length	Average Length (O_j_R_j_)
0.1	0.95	0.96	0.955	0.1	1.00	1.00	1.00
0.2	0.88	0.98	0.93	0.2	1.02	1.02	1.02
0.3	0.86	0.99	0.925	0.3	1.09	1.05	1.07
0.4	0.92	1.00	0.96	0.4	1.11	1.10	1.11
0.5	0.98	1.02	1.00	0.5	1.12	1.13	1.13
0.6	1.06	1.08	1.07	0.6	1.14	1.18	1.16
0.7	1.17	1.20	1.185	0.7	1.17	1.20	1.19
0.8	1.28	1.25	1.265	0.8	1.19	1.22	1.21
0.9	1.39	1.34	1.365	0.9	1.2	1.25	1.23
1.0	1.50	1.43	1.465	1.0	1.2	1.28	1.24
1.1	1.54	1.48	1.51	1.1	1.22	1.30	1.26
1.2	1.66	1.53	1.595	1.2	1.22	1.30	1.26
1.3	1.74	1.58	1.66	1.3	1.22	1.32	1.27
1.4	1.78	1.60	1.69	1.4	1.23	1.32	1.28
1.5	1.82	1.66	1.74	1.5	1.22	1.32	1.27
1.6	1.86	1.68	1.77	1.6	1.21	1.32	1.27
1.7	1.87	1.72	1.795	1.7	1.200	1.31	1.26
1.8	1.88	1.73	1.805	1.8	1.18	1.29	1.24
1.9	1.86	1.74	1.80	1.9	1.17	1.27	1.22
2.0	1.84	1.72	1.78	2.0	1.15	1.24	1.20
0	1.00	1.00	1.00				

**Table A4:** Cross-sectional profile of case S5 weld seam. (Unit: mm).

Height Upper (h_i_)	Left Length	Right Length	Average Length (O_i_R_i_)	Height Down (h_j_)	Left Length	Right Length	Average Length (O_j_R_j_)
0.1	0.70	0.65	0.675	0.1	0.68	0.64	0.66
0.2	0.70	0.68	0.69	0.2	0.68	0.64	0.66
0.3	0.75	0.70	0.725	0.3	0.65	0.64	0.645
0.4	0.76	0.72	0.74	0.4	0.63	0.68	0.655
0.5	0.78	0.74	0.76	0.5	0.60	0.74	0.67
0.6	0.79	0.76	0.775	0.6	0.60	0.82	0.71
0.7	0.81	0.78	0.795	0.7	0.60	0.88	0.74
0.8	0.84	0.82	0.83	0.8	0.62	0.96	0.79
0.9	0.86	0.86	0.86	0.9	0.63	1.02	0.825
1.0	0.89	0.89	0.89	1.0	0.69	1.06	0.875
1.1	0.92	0.92	0.92	1.1	0.77	1.10	0.935
1.2	0.92	0.93	0.925	1.2	0.85	1.14	0.995
1.3	0.92	0.95	0.935	1.3	0.89	1.17	1.03
1.4	0.92	0.97	0.945	1.4	0.93	1.21	1.07
1.5	0.92	1.02	0.97	1.5	0.95	1.24	1.095
1.6	0.92	1.05	0.985	1.6	0.98	1.26	1.12
1.7	0.92	1.06	0.99	1.7	0.99	1.28	1.135
1.8	0.93	1.08	1.005	1.8	1.03	1.32	1.175
1.9	0.95	1.10	1.025	1.9	1.05	1.32	1.185
2.0	0.96	1.12	1.04	2.0	1.06	1.32	1.19
0	0.68	0.66	0.67				

**Table A5:** Cross-sectional profile of case S6 weld seam. (Unit: mm).

Height Upper (h_i_)	Left Length	Right Length	Average Length (O_i_R_i_)	Height Down (h_j_)	Left Length	Right Length	Average Length (O_j_R_j_)
0.1	0.70	0.72	0.71	0.1	0.70	0.78	0.74
0.2	0.74	0.72	0.73	0.2	0.70	0.78	0.74
0.3	0.76	0.74	0.75	0.3	0.70	0.78	0.74
0.4	0.78	0.76	0.77	0.4	0.70	0.82	0.76
0.5	0.80	0.78	0.79	0.5	0.70	0.86	0.78
0.6	0.82	0.80	0.81	0.6	0.72	0.88	0.80
0.7	0.80	0.82	0.81	0.7	0.74	0.93	0.835
0.8	0.90	0.84	0.87	0.8	0.78	0.96	0.87
0.9	0.94	0.87	0.905	0.9	0.84	0.98	0.91
1.0	0.98	0.90	0.94	1.0	0.90	1.00	0.95
1.1	1.00	0.92	0.96	1.1	0.95	1.02	0.985
1.2	1.02	0.94	0.98	1.2	0.98	1.10	1.04
1.3	1.04	0.97	1.005	1.3	1.04	1.20	1.12
1.4	1.06	1.00	1.03	1.4	1.10	1.28	1.19
1.5	1.08	1.03	1.055	1.5	1.17	1.34	1.255
1.6	1.08	1.06	1.07	1.6	1.20	1.42	1.31
1.7	1.08	1.07	1.075	1.7	1.22	1.46	1.34
1.8	1.08	1.08	1.08	1.8	1.23	1.48	1.355
1.9	1.08	1.09	1.085	1.9	1.25	1.50	1.375
2.0	1.08	1.09	1.085	2.0	1.28	1.50	1.39
0	0.64	0.70	0.67				
